# Statistical analysis of arthroplasty register data

**DOI:** 10.3109/17453671003587168

**Published:** 2010-03-31

**Authors:** Jonas Ranstam, Otto Robertsson

**Affiliations:** Swedish Knee Arthroplasty Register, Department of Orthopedics, Lund University Hospital, Lund University, LundSweden

## Abstract

Data from arthroplasty registers are often analyzed using survival methods. Several methodological problems exist, for example relating to competing events, non-random censoring, non-proportional hazards and dependent observations. League tables and ranking of specific survival results leds to further methodological difficulties. Most of these problems are, however, well known and a number of methods for dealing successfully with the problems have been developed. These methods are usually accessible in commercially available statistical software packages.

The statistical analysis and reporting of data from arthroplasty registers can thus be improved. Development of arthroplasty register guidelines for statistical analysis could play an important role in making these registers even more useful.

## Background

The main purpose of arthroplasty registers is to detect failures as early as possible in order to provide warning of probable causes, such as inferior implants or surgical methods. The statistical analysis of joint replacement failure is generally performed using survival analysis techniques such as Kaplan-Meier analysis and Cox regression (Murray et al. 1993). A number of other methods, for example incidence density estimation and Poisson modeling, can be used for the same purpose, but in this paper we concentrate on the survival analysis techniques to avoid unnecessary complexity.

Two alternative techniques are available for describing cumulative survival (and failure): the Kaplan-Meier product limit method (Kaplan and Meier 1958) and the Cutler-Ederer actuarial life table method (Cutler and Ederer 1958). Both are available in commercial statistics packages such as SAS, STATA, and SPSS, and both have advantages and disadvantages.

In contrast to the Kaplan-Meier method, the Cutler-Ederer actuarial life table method is based on a series of fixed time intervals, which easily enables production of tables presenting the number of observations, events, withdrawals, survival estimates, etc. for specific intervals. This facilitates both the calculation and the presentation of results, but it has the disadvantages that the interval length is subjectively defined and the accuracy is slightly lower than with the Kaplan-Meier method.

Differences in survival are usually hypothesis-tested. Several methods have been developed, for example the log-rank test, which compares the entire survival experience and—when early events are of special interest—the generalized Wilcoxon test (Klein et al. 2001a). Several methods for calculation of confidence intervals for survival curves have been developed (Dorey et al. 1993).

Regression models are often used to adjust for differences in confounding variables, e.g. unbalanced distributions of age and sex. The proportional hazards model, also known as the Cox model (Klein et al. 2001b), is commonly used for this purpose in joint replacement analyses.

Relative to many other areas, however, survival analysis of joint replacements is problematic. Long follow-up times and low failure rates is one complicating factor, unfulfilled assumptions of the methods is a second one, and business interests and political ambitions in comparing the results of analyses is a third.

Some of these problems have already been extensively discussed in the literature and recommendations presented (Murray et al. 1993). In this paper, we aim to briefly describe a number of currently discussed methodological issues in survival analysis that have implications for the analysis of joint replacements and for which no general recommendations have yet been presented.

### Competing risks

The Kaplan-Meier method, the gold standard for estimating the survival of joint replacements (with results often presented in terms of cumulative revision risk), was developed for studying events that eventually occur in all subjects, i.e. death.

All patients will die eventually but all joint implants will not be revised. During an observation period some patients will undergo revision, some will die before revision becomes necessary, while others will reach the end of the observation period without either implant revision or death.

In this scenario, revision and death are two competing events, i.e. one event (death) hinders the other (revision) to occur. The Kaplan-Meier method is based on the assumption that competing events occur independently, e.g. that the risk of revision is the same in patients who die before the end of follow-up as in those living to the end of follow-up.

Assume, for example, that 5 patients with 1 hip replacement each are followed until a certain date. Before that date, 3 patients are revised while the 2 unrevised patients die before reaching the end of follow-up. The Kaplan-Meier method assumes that competing events do not exist, and that if the 2 deceased patients had been followed as long as the other 3 they would also have been revised. While only 60% of the patients have actually been revised, the estimated cumulative revision risk at end of follow-up is 100%.

This issue is also important when different implant failure endpoints are involved, for example when studying failures of different components such as the cup and the stem in hip arthroplasty. Here, the two endpoints represent competing events. A total revision caused by failure of one component excludes the possibility of the other component becoming a cause of revision. If the assumption of independence is not fulfilled, the results of the Kaplan-Meier method will be biased because it estimates the probability of an event occurring in a hypothetical population where competing events do not exist.

To get valid risk estimates, the probability of each competing event should be estimated. This can be achieved using the cumulative incidence estimator (Prentice et al. 1978) instead of the Kaplan-Meier method. Regression models, similar to the Cox model, for modeling effects of covariates on the cumulative incidence function have also been developed by Fine and Grey (1999), and by Klein and Andersen (2005).

It is important, however, to recognize that both approaches—Kaplan-Meier with Cox models and the cumulative incidence estimator with Fine and Grey or Klein and Andersen models—require the withdrawal of patients and events to be independent. For arthroplasty survival, this means that the risk of a patient dying or becoming lost to follow-up is unrelated to the risk of becoming revised. The term informative censoring is used to describe a relation between the risks of withdrawal and events that may bias the results of the survival analysis (Scharfstein and Robins 2002).

It has been suggested that competing risk situations are common in orthopedic research and that the resulting bias may be substantial. However, appropriate statistical methods are available to address these problems (Biau et al. 2007).

### Proportional hazards

The Cox model is often used when comparing survival estimates for different implant models, patient categories, hospitals, etc. It enables estimation of crude and adjusted relative risks representative of the entire survival experience. The validity of these relative risks relies, however, on the assumption of proportional hazards, i.e. that the relative effects of covariates are constant over time (Collet 1994)—e.g. for 2 types of implants, the proportions of early and late revisions are the same, see Figure.

**Figure:**
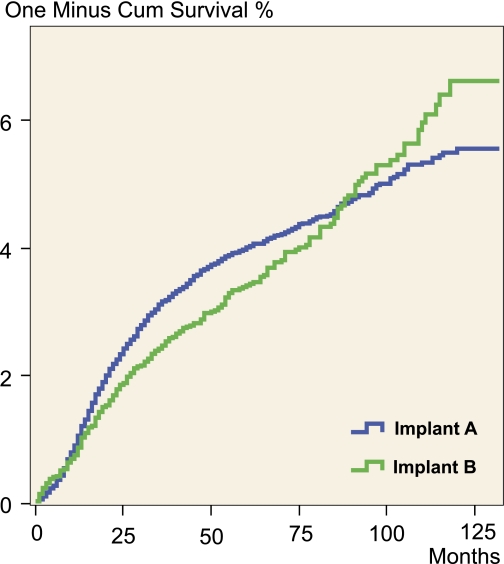
Cumulative revision rate of two fictitious prosthesis types characterized by non-proportional hazards.

Several ways to check this assumption have been devised, one of the most important being based on the test of Schoenfeld's partial residuals for a covariate (Schoenfeld 1982). This test is incorporated in most statistical software packages that include Cox regression.

Non-proportional hazards are probably not uncommon in orthopedic research, and several methods such as stratification, piecewise Cox modeling, and incorporation of time-dependent variables have been developed to account for non-proportionality. However, many published Kaplan-Meier survival curves, calculated from data that are also analyzed using the Cox model, show that not all investigators recognize the requirement of proportional hazards. Non-proportional hazards is, for example, indicated by two survival curves crossing each other.

It is sometimes argued that a relative risk estimated with non-proportional hazards represents the average relative risk during follow-up, but this is incorrect. The relative risk is over- or underestimated depending on whether the risks increase or decrease over time, because there is more information on failure risk at the beginning of follow-up than at the end. The testing power of the relative risk is also reduced (Schemper 1992).

A Cox model with weighted estimation providing valid average relative risks with non-proportional hazards has been developed (Schemper et al. 2009). The advantage of this model is that it provides valid results without inclusion of additional parameters.

Diagnosing non-proportional hazards and adjusting the analysis for this is a vital but often neglected part of performing a survival analysis with the Cox model. This issue clearly needs to be given more attention.

### Bilaterality

Another important assumption, on which survival analysis and many other statistical methods are based, is that analysis units are independent. This assumption is not fulfilled when subjects contribute more than one observation each to the statistical analysis, for example from bilateral or multilateral implants. A patient with 2 implants may, for example, develop infection in both after having one episode of sepsis.

In a statistical analysis of independent observations, only one source of variation is included: variation between subjects. When analyzing bilateral observations, two sources of variation must be taken into account: variation between subjects and variation between implants within subjects. As such an analysis includes estimation of both fixed (mean values) and random (variance) effects, the analysis technique is known as mixed effects analysis.

The Cox model can be used for this purpose by extending it to include a subject-specific frailty component, common for bilateral observations from the same subject but varying between subjects (Hougaard 2000). This frailty component is often assumed to be gamma distributed. The model is then described as a shared gamma frailty model.

It has been investigated if the inclusion of bilateral observations has any effect on the results from survival analyses of hip and knee implants (Ripatti and Palmgren 2000, Schwarzer et al. 2001, Robertsson and Ranstam 2003, Lie et al. 2004). The investigations all suggest that bilaterality has a negligible effect on survival estimates.

Studies on other types of implants, with different endpoints, study settings, follow-up times etc. may be less robust against dependent observations (e.g. multiple finger arthroplasties or when using infection as endpoint).

A recent systematic review has shown that disregarding dependency is common in orthopedic publications (Bryant et al. 2006). This speaks for the importance of awareness of the problem.

### Hospital comparisons

Swedish registries are required to provide information on hospital-specific revision risks as part of open national comparisons initiated by governmental authorities. Registers in other countries may face similar challenges. Comparison of revision risks between hospitals is complicated by several factors.

First, the characteristics of patients, which are often presented as a case-mix, vary naturally between hospitals. Ideally, the influence on the revision rates from the case-mix should be removed in the statistical analysis, because the aim of the comparisons is to focus on hospital achievements that can be improved.

If all factors determining the case-mix were known and measured, it might be possible to adjust for their differential effects using a Cox model. This is not, however, possible in practice as not all factors are known or measured. The Cox model is still used to adjust for known and measured differences in case-mix, such as age, sex, and diagnosis, but this adjustment may not be perfect, leaving residual confounding effects from differences in other patient and treatment factors.

Secondly, the comparisons are further complicated by low failure rates, failures occurring a long time after the primary operation, and large variation in the number of operations per hospital. The amount of information available for estimation of a hospital-specific revision risk varies substantially between hospitals. Hospitals with few primary operations have a greater probability of—by chance—showing extremely high or low revision risks than hospitals with many primary operations. Estimates based on few patients are simply more uncertain.

To take differences in operative volumes into account, the above-described frailty model can also be used when comparing hospital-specific revision risks. The subject-specific frailty component is then replaced by a hospital-specific one. The hospital-specific revision risks, estimated by the frailty component, take the varying amount of information available at the hospitals into account. The resulting risk estimates are therefore shrunken as compared to those from a traditional Cox model, and that reduces the problem of over-interpreting randomly high and low risks caused by differences in operative volumes (Robertsson et al. 2006).

### Ranking

The government initiative of open comparisons described earlier includes publication of tables that are commonly used to rank hospitals. This is, however, an uncertain method to compare hospital-specific risks. Apart from the problems described above related to case-mix and extreme risks, sampling and measurement uncertainty have profound effects on the interpretation of ranks.

Published league tables often include confidence intervals for the hospital-specific risks that are ranked, but this provides little information on rank uncertainty. However, confidence intervals for ranks can also be calculated. The calculation is not straightforward; it requires computer simulation. Even so, to avoid misleading the reader it is important to inform him or her about the reliability of presented ranks (Ranstam et al. 2008a). The Swedish Knee Arthroplasty Register also routinely presents this kind of information, see Table.

There are registration errors in most registers, and the magnitude of these is often not known in detail. Joint registers typically define revision by linking of patients' operation records. If mis-registration of an item of information prevents a patients' records from being linked, the revision will not be identified but will be treated as a second primary operation.

The consequence of such registration errors on hospital ranks can be formidable. A simulation study using published data on hip arthroplasties showed, for example, that hospital-specific ranking of revision risks was unreliable already at 2–3% misclassified revisions (Ranstam et al. 2008b). Furthermore, implant survival is usually the result of surgeries performed over many years. Thus, it is not at all certain that the circumstances that caused any observed differences exist at the time of publication.

**Table:** Extract from annual report of the Swedish Knee Arthroplasty Register (SKAR) showing uncertainty indiction for ranking. The extract is published with approval from SKAR

Code	Unit	No. TKA	No. revised	RR	95% CI	Rank	95% CI
21001	Linköping	482	2	0.33	0.15–0.73	3	1–27
21014	Motala	1683	8	0.38	0.21–0.66	4	1–22
10484	Sabbatsbergs närsjh	704	5	0.38	0.20–0.72	4	1–26
52012	Alingsås	851	5	0.45	0.24–0.87	8	1–39
56010	Västerås	457	3	0.49	0.24–1.03	10	1–50
62011	Örnsköldsvik	913	8	0.49	0.28–0.87	10	1–38
22010	Jönköping	849	8	0.51	0.29–0.90	11	2–40
64011	Lycksele	352	2	0.52	0.23–1.14	12	1–58
22012	Värnamo	804	7	0.55	0.30–1.00	13	2–48
50010	östra sjukhuset	884	9	0.56	0.33–0.97	15	2–45
53011	Lidköping	730	7	0.58	0.32–1.04	15	2–52
65014	Kalix	164	1	0.58	0.24–1.39	15	1–69
12010	Enköping	1006	9	0.59	0.35–1.02	16	3–50
53010	Falköping	770	8	0.60	0.34–1.05	17	3–51
42011	Varberg	1137	13	0.60	0.37–0.98	17	4–47
50001	Sahlgrenska	447	5	0.62	0.32–1.19	18	3–59
RR: Relative risk of revision for units							

Clearly, ranking is not a good way to describe hospital achievements.

### Guidelines for reporting of joint survival

In summary, survival analysis has been used extensively for reporting of joint register data. A number of methodological problems have been identified, while theoretical developments have brought forward methods to deal successfully with many of them. These new methods have also become accessible in a number of commercially available statistical software packages.

Joint registries were initiated to gain knowledge on the outcome of joint arthroplasty and how it was affected by factors related to the patients, implants, and surgical methods. While providing important information that can be used as a basis for improvement, their findings have also had a pronounced effect on providers of individual implants and lately also on hospitals. The high profile of their findings may partly explain the reluctance of the registries to adopt new methods; instead, the retain ones that are widely used by others and that are accepted in the orthopedic literature.

Improvement of statistical analysis and reporting of data is a matter of communicating information and altering practice, however. We believe that the development of common joint register guidelines could play an important role by enabling coordination of the choice of modern statistical methods, selections, endpoints, and mode of reporting. This would make the joint registers even more useful.

International joint replacement registry associations may be the right forum for such an initiative.
